# Acoustic characterization of a neonate skull using a clinical MR-guided high intensity focused ultrasound system for pediatric neurological disorder treatment planning

**DOI:** 10.1186/2050-5736-3-S1-P14

**Published:** 2015-06-30

**Authors:** Elodie Constanciel Colas, Adam Waspe, Charles Mougenot, Thomas Looi, Samuel Pichardo, James Drake

**Affiliations:** 1 Centre for Image Guided Innovation and Therapeutic Intervention, Toronto, Canada; 2 Philips Healthcare Canada, Toronto, Canada; 3 Thunder Bay Regional Research Institute, Thunder Bay, Canada

## Background/introduction

Transcranial MR-guided Focused Ultrasound (TcMRgFUS) treatments are now clinically performed on adult patients for brain tumor or essential tremor therapies. However, no application has been proposed for children despite their thinner skull being less of an acoustic barrier and the presence of a fontanelle on neonates, which could constitute a natural acoustic window for the transmission of ultrasound waves. As there is minimal literature data on the attenuation and speed-of-sound of the skull in neonatal patients, the aim of this study was to perform the acoustic characterization of a neonate skull.

## Methods

A 0.2 mm needle acoustic hydrophone was placed in a tank of degassed water and aligned to the geometric focus of a clinical HIFU transducer (Philips Sonalleve). The signals of the 256 elements of the phased array transducer were acquired as a baseline measurement using this hydrophone. A degased cadaveric neonate skull was then placed inside the tank between the hydrophone and the transducer. Acquisitions were performed for different angular orientations of the skull according to the sagittal and coronal axes in the range of ± 15°. Insertion losses (IL) and time-of-flight (TOF) delays due to the skull and the fontanelle were deducted from these measurements performed at 1 MHz and 1.2 MHz.

## Results and conclusions

When the acoustic axis of the transducer was normal to the fontanelle, the average IL and TOF delay due to the fontanelle were respectively 0.9 ± 0.8 dB and -0.09 ± 0.02 μs at 1 MHz. At 1.2 MHz, the IL decreased at 0.5 ± 0.5 dB and the TOF delay remained the same. Rotations of the skull around the coronal axis giving access to the parietal and frontal bones, their average IL were respectively 1.6 ± 0.7 dB and 2.9 ± 1.1 dB at 1MHz and 1.2 ± 0.5 dB and 2.0 ± 0.7 dB at 1.2 MHz. Rotations of the skull around the sagittal axis allowed the measurements of the properties of the bones on the left and the right sides of the skull. The average IL of the bones on the left side was 2.0 ± 0.8 dB at 1MHz and 1.2 MHz. The average IL of the bones on the right side was 2.1 ± 0.7 dB at 1MHz and at 1.7 ± 0.4 dB at 1.2 MHz. The average TOF delay due to the skull bones was globally the same and was -0.17 ± 0.02 μs at 1 MHz and 1.2 MHz. For the two studied frequencies, low IL and TOF delay values were obtained for the fontanelle and to a lesser extent for the skull bones. Slightly higher IL were obtained at 1 MHz compared to 1.2 MHz. These results confirm that the fontanelle can act as an acoustic window for future TcMRgFUS applications. They also emphasise the potential of this technology for neurological disorder treatments on neonate patients.

**Figure 1:**
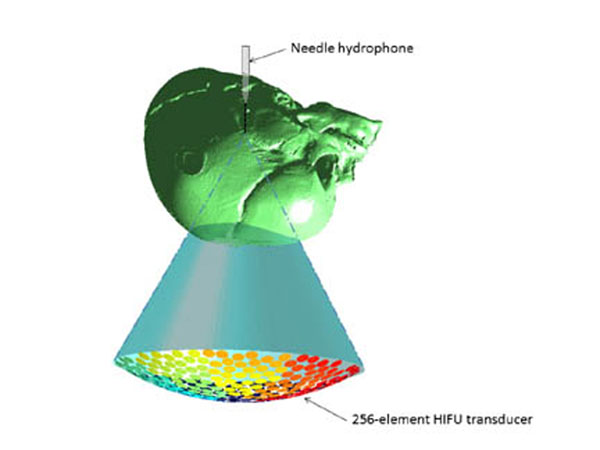
Computer rendering showing the skull and hydrophone positioning relative to the HIFU transducer beam

